# Combination effect between gut microbiota and traditional potentially modifiable risk factors for first-ever ischemic stroke in Tujia, Miao and Han populations in China

**DOI:** 10.3389/fnmol.2022.922399

**Published:** 2022-10-25

**Authors:** Na Zhang, Haoren Wang, Xiaolei Wang, Mengyuan Tian, Yong Tian, Qi Li, Chengcai Liang, Xiaowei Peng, Jian Ding, Xinrui Wu, Hongzhuan Tan

**Affiliations:** ^1^Hunan Provincial Key Laboratory of Clinical Epidemiology, Department of Epidemiology and Health Statistics, Xiangya School of Public Health, Central South University, Changsha, China; ^2^Department of Geriatric Rehabilitation, Hunan Provincial Rehabilitation Hospital, Changsha, China; ^3^Department of Neurology, The First Affiliated Hospital of Jishou University, Xiangxi, China; ^4^Department of Planned Immunity, Xiangxi Center for Disease Prevention and Control, Xiangxi, China; ^5^Jishou University School of Medicine, Xiangxi, China

**Keywords:** ischemic stroke, gut microbiota, additive interaction, risk factor, prevention

## Abstract

China has had explosive growth in ischemic stroke (IS) burden with significant ethnic and geographic disparities. The aim of this study was to explore the possible combination effect between gut microbiota and traditional potentially modifiable risk factors for IS among two ethnic minorities (Tujia and Miao) and the Han population. Herein, we first used the 16 S rRNA sequencing to compare the gut microbial compositions of 82 patients with first-ever IS vs. 82 normal controls (NCs) among Han, Tujia, and Miao people between 1 May 2018 and 30 April 2019, from Xiangxi Tujia and Miao Autonomous Prefecture in China. An additive model was used to study the interaction between traditional risk factors and gut microbiota with R software. Linear discriminant analysis (LDA) and LDA effect size (LEfSe) results showed that the identified key gut microbiota's taxonomic composition varied in different ethnicity between the IS patients and NCs. Furthermore, families *Lactobacillaceae, Enterococcaceae, Streptococcaceae*, and *Enterobacteriaceae* were found to be positively correlated with high-risk factors and negatively correlated with preventive factors in the IS patients, but families *Ruminococcaceae* and *Lachnospiraceae* were just the opposite in the NCs. There were additive interactions between traditional risk factors (systolic blood pressure, diastolic blood pressure, and high-sensitive C-reactive protein) and family *Enterococcaceae* for first-ever IS with the attributable proportion due to the interaction was 0.74, 0.71, and 0.85, respectively; and the synergy index was 4.45, 3.78, and 7.01, respectively. This preliminary but promising study showed that the gut microbiota disturbances may potentially interact to IS with different ethnic host's traditional risk factors.

## Introduction

Ischemic stroke (IS) occurs when a vessel supplying blood to the brain is obstructed, and it constitutes 69.6% and 77.8%, respectively, in total stroke incidence and prevalence in China (Wang et al., 2017). The INTERSTROKE investigators found ten potentially modifiable risk factors, including previous history of hypertension or blood pressure of 140/90 mmHg or higher, regular physical activity, apolipoprote in (Apo) B/ApoA1 ratio, diet, waist-to-hip ratio, phychosocial factors, current smoking, cardiac causes, alcohol consumption, and diabetes mellitus, that were collectively associated with about 91.5% of the population attributable risk (PAR) of IS in each major region of the world, among ethnic groups, in men and women, and in all ages (O'Donnell et al., 2016). Despite significant progress in the prevention and treatment strategies in the past two decades, the Global Burden of Disease 2017 Study reported that stroke is still the leading cause of death and disability-adjusted life years at the national level in China and the third cause of global years of life lost (O'Donnell et al., 2016; GBD 2017 Causes of Death Cllaborators., 2018; Zhou et al., 2019). Hence, IS has become one of the most important disease burdens and a major public health problem in China, even in the world.

Accumulating evidence demonstrated that gut microbiota can exert considerate influence over the gastrointestinal physiology, immune function, and even mental wellbeing through the microbiota-gut-brain axis (Sharon et al., 2016; Dinan and Cryan, 2017; Fung et al., 2017). Although the functional significance of the symbiotic relationship between host and microbe especially in the context of brain health has not been fully elucidated, several tools and animal models have been invaluable in allowing the scientific research community to constantly narrow the gaps in our understanding of the microbiota-gut-brain axis. Gut permeability, gastrointestinal motility, and microbiota composition are all drastically altered in mouse models of stroke (Houlden et al., 2016; Singh et al., 2016; Stanley et al., 2016). Furthermore, fecal microbiota transplantation (FMT) into germ-free (GF) mice from a cerebral ischemia model transmitted functional deficits of proinflammatory T-lymphocyte (Th1 and Th17 T_helper_ phenotype) trafficking and the ischemia-induced cerebral lesion volume (Singh et al., 2016). To date, only a few studies have preliminarily explored the gut microbiota in IS patients. In a Chinese patient group presenting with stroke or a transient ischemic attack, fecal abundance of three major commensal microbes (*Bacteroides, Prevotella*, and *Faecalibacterium*) were severely depleted, with a concomitant enhancement in abundance of opportunistic pathogens such as *Enterobacter, Megasphaera*, and *Desulfovibrio* (Yin et al., 2015). Moreover, patients presenting with severe stroke had a greater abundance of *Proteobacteria* over those with milder stroke.

The INTERSTROKE investigators also found that there are regional and ethnic variations in the effect of risk factors, which was related to differences in risk factors prevalence among regions that may be contribute to worldwide variations in the frequency of IS and support the development population-specific measures to prevent IS (O'Donnell et al., 2016). China is a unified multi-ethnic country with 56 ethnic groups, of which the Han is the largest group, and the other 55 ethnic groups are referred to as ethnic minorities. According to the sixth national population census in 2010, the Miao (9.43 million) and Tujia (8.35 million) minorities were ranked as the fifth and seventh largest minority groups, respectively. Our previous epidemiological studies have found that there was an additive interaction between the ApoB/ApoA1 ratio and ethnicity in the Tujia and Miao populations with first-ever ischemic stroke (the relative excess risk due to the interaction was 5.75) (Zhang et al., 2021), but few reliable data are available to identify the differences in the gut microbiota of IS among the people of Tujia, Miao, and Han. As the data were limited in previous studies, this study aims to study the differences among Tujia, Miao, and Han populations in the gut microbiota of IS patients in Xiangxi Tujia and Miao Autonomous Prefecture (Xiangxi), and to study the association between gut microbiota and traditional risk factors of IS patients for providing new insights into the risk factors, prevention, and management strategies of IS among the people of Tujia, Miao, and Han ethnicities.

## Materials and methods

### Study population

Participants were recruited between 1 May 2018 and 30 April 2019, from Xiangxi, located in western Hunan Province and adjacent to Hubei and Guizhou Provinces, where people of Tujia and Miao constitute 80% of the total population of 2.98 million. All the participants are residents who had lived in Xiangxi more than 5 years. All patients who admitted to the First Affiliated Hospital of Jishou University and all the eight county people's hospitals of Xiangxi, within 5 days of symptoms onset and 72 h of hospital admission, with a discharge diagnosis of first-ever IS, were selected as a case group. IS was diagnosed with the WHO clinical criteria (Hatano, 1976). A CT or MRI of the brain was completed within 1 week of presentation. All patients also underwent a range of blood tests, a chest radiograph, and an electrocardiography (ECG) at admission. According to the Trial of Org 10,172 in Acute Stroke Treatment (TOAST) criteria (Adams et al., 1993), IS was divided into the following: (1) large-artery atherosclerosis, (2) cardioembolism, (3) small-vessel occlusion, (4) stroke of other determined etiology, and (5) stroke of undetermined etiology. The disease severity was estimated by National Institutes of Health Stroke Scale (NIHSS) score on admission by the physicians. For patients unable to communicate adequately, proxy respondents were used. We defined a valid proxy respondent as a spouse or first-degree relative who was living in the same home or was aware of the participant's previous medical history and present treatments. Exclusion criteria: (1) patients unable to communicate and without a valid surrogate respondent; (2) subdural hemorrhage, tumor, and brain abscess (i.e., non-vascular causes); (3) current hospitalization for acute coronary syndrome/myocardial infarction; and (4) unable to get consent from the subject or the surrogate. Community-based normal controls (NCs) were all from the eight counties and had no history of stroke. Each control was matched for sex, ethnicity, and age (±5 years) for every case. Exclusion criteria for controls are identical to those described for cases. Species accumulation boxplot was used to validate the sample size (Colwell et al., 2004). Interviewer bias may result when knowledge of case or control status influences the manner questions asked or indirectly influences interviewee response. To overcome this potential bias, interviewers are trained to obtain information in a standardized fashion. Recall bias may result when the presence or absence of a medical condition may influence patient's or caregiver's ability to recall events. The objective evidence of risk factors (e.g., history of allergies and history of antibiotic administration) is sought, whenever possible.

### Sample collection and processing

Fasting blood samples (10 mL) were taken from IS patients and NCs within 72 h of recruitment, separated by centrifugation, and frozen at −80°C immediately after processing. Samples were shipped in packaging incorporating dry ice-cooling agents by courier from every site to a blood storage site, where they were stored at −80°C. Total cholesterol (TC), triglyceride (TG), low-density lipoprotein cholesterol (LDL-C), high-density lipoprotein cholesterol (HDL-C), apolipoprotein B (ApoB), ApoA1, fasting plasma glucose (FPG), high-sensitive C-reactive protein (hs-CRP), and homocysteine (Hcy) concentrations were measured in the laboratory of the Dian Diagnostics Group Co. Ltd with Beckman Coulter AU680 Clinical Chemistry Analyzer and Beckman reagents (Beckman Coulter, Brea, CA, USA). All the participants have not taken antibiotics, probiotics, and prebiotics in the 3 months prior to the feces collection. Feces were collected at hospital or home. The feces were collected according to the instruction and delivered immediately at low temperatures. The frozen feces were shipped using dry ice to Xiangxi Center for Disease Prevention and Control. Once received, fecal samples were divided into three parts of 200 mg and stored at −80°C until extraction.

### DNA extraction, PCR amplification, and Ion S5^TM^ XL sequencing

CTAB method was used to extract total genome DNA from samples according to the DNA extraction kit (#DP328, Tiangen Company, Beijing, China). The concentration and purity of the extracted bacterial DNA were detected using Qubit^®^ 2.0 Fluorometer (Thermo Scientific, USA). The 16S rRNA gene V4 regions were amplified used specific primers 515F (5'-GTGCCAGCMGCCGCGGTAA-3') and 806R (5'-GGACTACHVGGGTWTCTA AT-3') with the barcode. All PCR reactions were carried out in 30 μL reactions with 15 μL of Phusion^®^ High-Fidelity PCR Master Mix (New England Biolabs). The mixture of PCR products was purified with GeneJET^TM^ Gel Extraction Kit (Thermo Scientific). Sequencing libraries were generated using Ion Plus Fragment Library Kit 48 rxns (Thermo Scientific) following manufacture's recommendations. The library quality was assessed on the Qubit^®^ 2.0 Fluorometer (Thermo Scientific). At last, the library was sequenced on the Ion S5^TM^ XL platform and 400 bp/600 bp single-end reads were generated.

### 16S rRNA gene sequence analysis

Quality filtering on the raw reads was performed under specific filtering conditions to obtain the high-quality clean reads according to the Cutadapt (V1.9.1, https://cutadapt.readthedocs.io/en/stable/) quality-controlled process (Martin, 2011). The reads were compared with the reference database (Silva Database, https://www.arb-silva.de/) using UCHIME algorithm (http://www.drive5.com/usearch/manual/uchime_algo.html) to detect chimera sequences (Edgar et al., 2011; Quast et al., 2013), and then the chimera sequences were removed. Then, the Clean Reads finally obtained.

Sequence analyses were performed by Uparse software (Uparse v7.0.1001, http://drive5.com/uparse/) (Edgar, 2013). Sequences with ≥97% similarity were assigned to the same operational taxonomic units (OTUs). Representative sequence for each OTU was screened for further annotation. For each representative sequence, the Silva 138.1 was used based on Mothur algorithm to annotate taxonomic information (Quast et al., 2013). In order to study phylogenetic relationship of different OTUs and the difference of the dominant species in different samples (groups), multiple sequence alignments were conducted using the MUSCLE software (V3.8.31, http://www.drive5.com/muscle/) (Edgar, 2004).

Participants' metadata was obtained about ethnicity, sex, age, body mass index (BMI), systolic blood pressure (SBP), diastolic blood pressure (DBP), TC, TG, LDL-C, HDL-C, ApoB/ApoA1 ratio, FPG, hs-CRP, and Hcy. All alpha diversity indices in our samples were calculated with QIIME (V1.7.0) and displayed with R. Wilcoxon rank sum test was used to perform hypothesis test of alpha diversity. Principal coordinates analysis (PCoA) was displayed by WGCNA package, STAT packages, and ggplot2 package in R. Analysis of similarities (ANOSIM) was displayed by function anosim of vegan package in R. The key bacterial taxa responsible for discrimination between NCs and IS patients were identified using linear discriminant analysis (LDA) and LDA effect size (LEfSe). LEfSe is available as a Galaxy module, a Conda formula, a Docker image, and included in bioBakery (VM and cloud) (Segata et al., 2011).

### Statistical analysis

Continuous variables were described as mean ± standard deviation (SD) if they were normally distributed or median (interquartile range, IQR) if not normally distributed, and compared using independent samples *t*-test or Mann-Whitney *U-*test. Spearman's rank correlation analysis between gut microbiota and traditional risk factors was constructed by corr.test in the psych package from R, after which the visualization work was done by the pheatmap function in pheatmap package. Three measures of biological interaction—relative excess risk of interaction (*RERI*), attributable proportion due to interaction (*API*), and synergy index (*SI*)—with corresponding 95% confidence intervals (CIs) were calculated in R (Andersson et al., 2005; Källberg et al., 2006). The *RERI* or *API* equal to 0 or *SI* equal to 1 were defined as no interaction (Andersson et al., 2005). Statistical analyses and graphics were produced with IBM SPSS Statistics for Windows, V23.0 (Armonk, NY: IBM Corp, Released 2015) and R for Windows (Version 4.0.3). All statistical tests of hypotheses are two-sided. Statistical significance level was set at *P* < 0.05.

## Results

### Basic information of recruited subjects

The study included 82 IS patients and 82 NCs, age ranged from 41 to 79 years old. The TOAST classification of IS patients are: large-artery atherosclerosis (*n* = 34, 41.5%); cardioembolism (*n* = 1, 1.2%); and small-vessel occlusion (*n* = 47, 57.3%). The NIHSS score of the IS patients were: 0 as no stroke symptoms (*n* = 10, 12.2%); 1–4 as minor stroke (*n* = 48, 58.5%); 5–15 as moderate stroke (*n* = 24, 29.3%). The mean values of BMI, SBP, DBP, TC, TG, LDL-C, HDL-C, ApoB/ApoA1 ratio, FPG, hs-CRP, and Hcy were 25.58 ± 3.84 kg/m^2^, 148.28 ± 20.63 mmHg, 91.77 ± 13.19 mmHg, 4.50 ± 0.94 mmol/L, 1.72 ± 1.00 mmol/L, 2.68 ± 0.76 mmol/L, 1.19 (0.37) mmol/L, 0.81 (0.38), 5.99 (2.57) mmol/L, 5.00 (0.00) mg/L, and 14.30 (7.20) μmol/L, respectively, in IS patients. The mean values of the same clinical indexes were 24.47 ± 3.33 kg/m^2^, 134.12 ± 18.73 mmHg, 81.10 ± 10.44 mmHg, 4.84 ± 1.07 mmol, 1.48 ± 0.81 mmol/L, 2.41 ± 0.72 mmol/L, 1.40 (0.57) mmol/L, 0.64 (0.27), 5.34 (1.37) mmol/L, 1.02 (1.72) mg/L, and 15.15 (6.85) μmol/L, respectively, in NCs. Differences of BMI, SBP, DBP, TC, LDL-C, HDL-C, ApoB/ApoA1 ratio, FPG, and hs-CRP between the two groups were both statistically significant. The detailed ethnicity and sex characteristics of the two groups are shown in Table 1.

**Table 1:** Demographic and traditional risk factors of recruited subjects.

**Demographic and traditional risk factors**	**IS patients (*N* = 82)**	**NCs (*N* = 82)**	***T***	***P***
Tujia/Miao/Han	30/27/25	30/27/25	–	–
Male/Female	49/33	49/33	–	–
Age (years)	62.57 ± 9.26	63.38 ± 9.34	−0.554	0.580
Body mass index (kg/m^2^)	25.58 ± 3.84	24.47 ± 3.33	1.980	0.049
Systolic blood pressure (mmHg)	148.28 ± 20.63	134.12 ± 18.73	4.602	0.000
Diastolic blood pressure (mmHg)	91.77 ± 13.19	81.10 ± 10.44	5.745	0.000
Total cholesterol (mmol/L)	4.50 ± 0.94	4.84 ± 1.07	−2.144	0.034
Triglyceride (mmol/L)	1.72 ± 1.00	1.48 ± 0.81	1.687	0.094
Low-density lipoprotein cholesterol (mmol/L)	2.68 ± 0.76	2.41 ± 0.72	2.337	0.021
High-density lipoprotein cholesterol (mmol/L)	1.19 (0.37)	1.40 (0.57)	–	0.000
Apolipoprotein B (ApoB)/ApoA 1 ratio	0.81 (0.38)	0.64 (0.27)	–	0.000
Fasting plasma glucose (mmol/L)	5.99 (2.57)	5.34 (1.37)	–	0.002
High-sensitive C-reactive protein (mg/L)	5.00 (0.00)	1.02 (1.72)	–	0.000
Homocysteine (μmol/L)	14.30 (7.20)	15.15 (6.85)	–	0.854

Date are mean ± SD or median(IQR). *P* values derived from the comparison between IS patients and NCs using independent samples *t*-test or Mann-Whitney *U* test. IS, ischemic stroke; NCs, normal controls; Tujia, the Tujia people; Miao, the Miao people; Han, the Han people; SD, Standard deviation; IQR, Interquartile range.

### Differences of gut microbiota composition between normal controls and ischemic stroke patients

**Figure 1:**
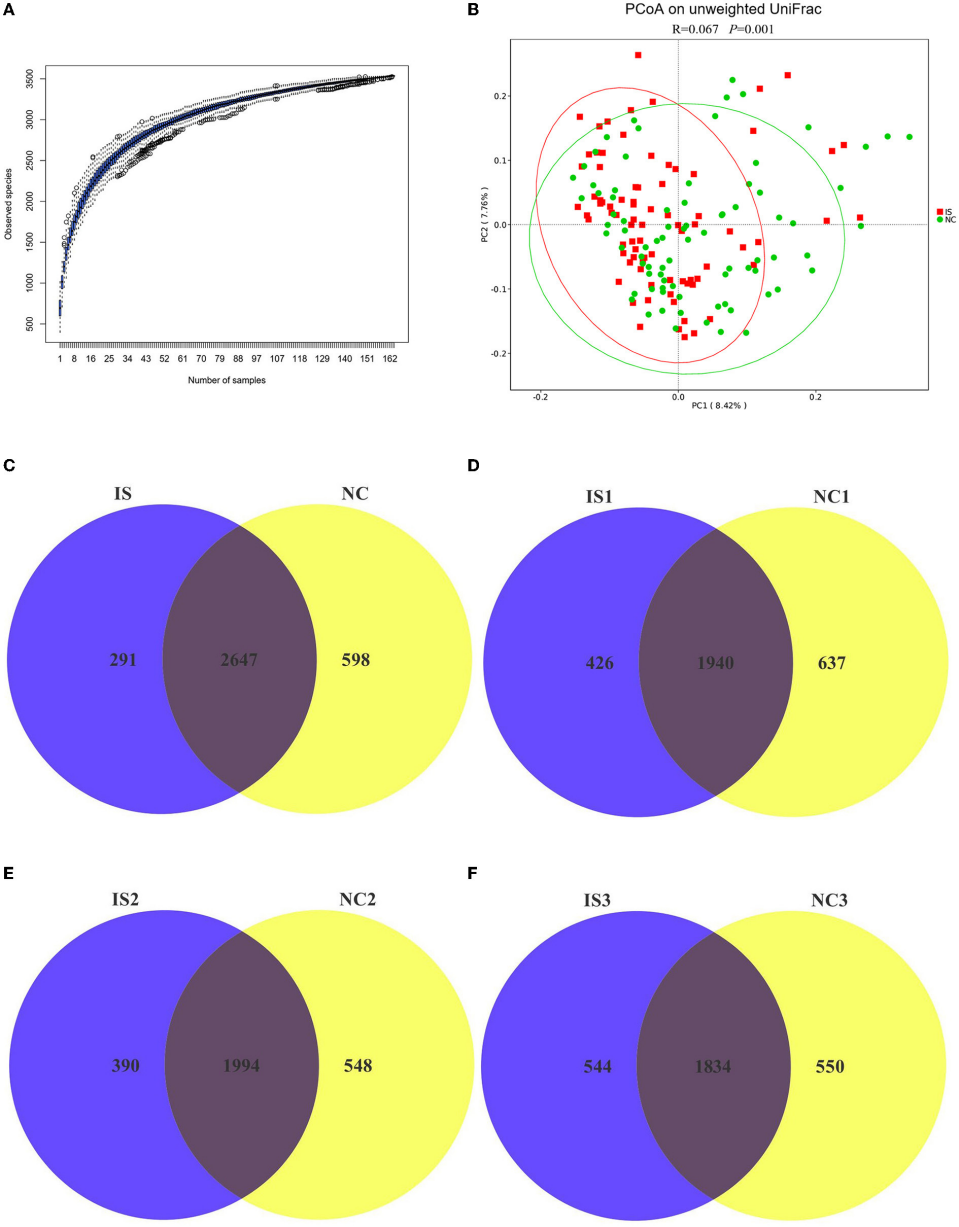
The differences of OTUs between normal controls (NCs) and ischemic stroke (IS) patients in the entire population and for each ethnic people (Tujia, Miao, and Han) according to the 16S rRNA data. The IS patients of the Tujia, Miao, and Han people were indicated with IS1, IS2, and IS3, respectively. The NCs of the Tujia, Miao, and Han people were indicated with NC1, NC2, and NC3, respectively. **(A)** Species accumulation boxplot of the entire population. **(B)** Principal coordinate analysis (PCoA) of the microbiota based on the unweighted UniFrac distance metrics for NCs and IS patients. ANOSIM, *R* = 0.067, *P* = 0.001. **(C–F)** Venn diagram of the observed OTUs in NCs and IS patients in the entire population and for each ethnic people (Tujia, Miao, and Han).

In total, we identified 12,808,213 high-quality reads in 164 fecal samples with an average length of 253. The species accumulation boxplot is an effective method to evaluate whether the sample size is sufficient or not. The boxplot is tended to be flat (Figure 1A), indicating that the sequencing sample size was ample, and the results were reliable. The top 10 in relative abundance of gut microbiota (in the level of phylum, class, order, family, genus, and species, respectively) between NCs and IS patients among Tujia, Miao, and Han ethnicity are shown in Supplementary Figure 1. Detailed information about alpha diversity indexes between NCs and IS patients among Tujia, Miao, and Han ethnicity are shown in Supplementary Figure 2. A PCoA was performed to investigate the extent of the similarity of the microbial communities between NCs and IS patients based on unweighted UniFrac distance metrics (Figure 1B); the analysis indicated that the microbiota composition of IS patients was more heterogeneous and significantly different from NCs. These reads could be annotated and classified into 3,536 OTUs in the entire population by the Silva 138.1 Database, and 90.27% of OTUs were annotated in the level of family. So the follow-up analysis and discussion were based on the family level of gut microbiota. In total, 2,647 OTUs were shared between the NCs and IS groups, while 598 and 291 OTUs were unique to NCs and IS patients, respectively (Figure 1C). As shown in Figure 1D, Venn diagram displayed 637 unique OTUs in the NCs and 426 unique OTUs in the IS patients, and 1,940 OTUs were shared by both groups of the Tujia people. In the Miao people, 1,994 OTUs were shared between NCs and IS patients, while 548 and 390 OTUs were unique to NCs and IS patients, respectively (Figure 1E). In the Han people, 1,834 OTUs were shared between NCs and IS patients, while 550 and 544 OTUs were unique to NCs and IS patients, respectively (Figure 1F).

### The microbial signatures responsible for distinguishing ischemic stroke patients from normal controls

**Figure 2:**
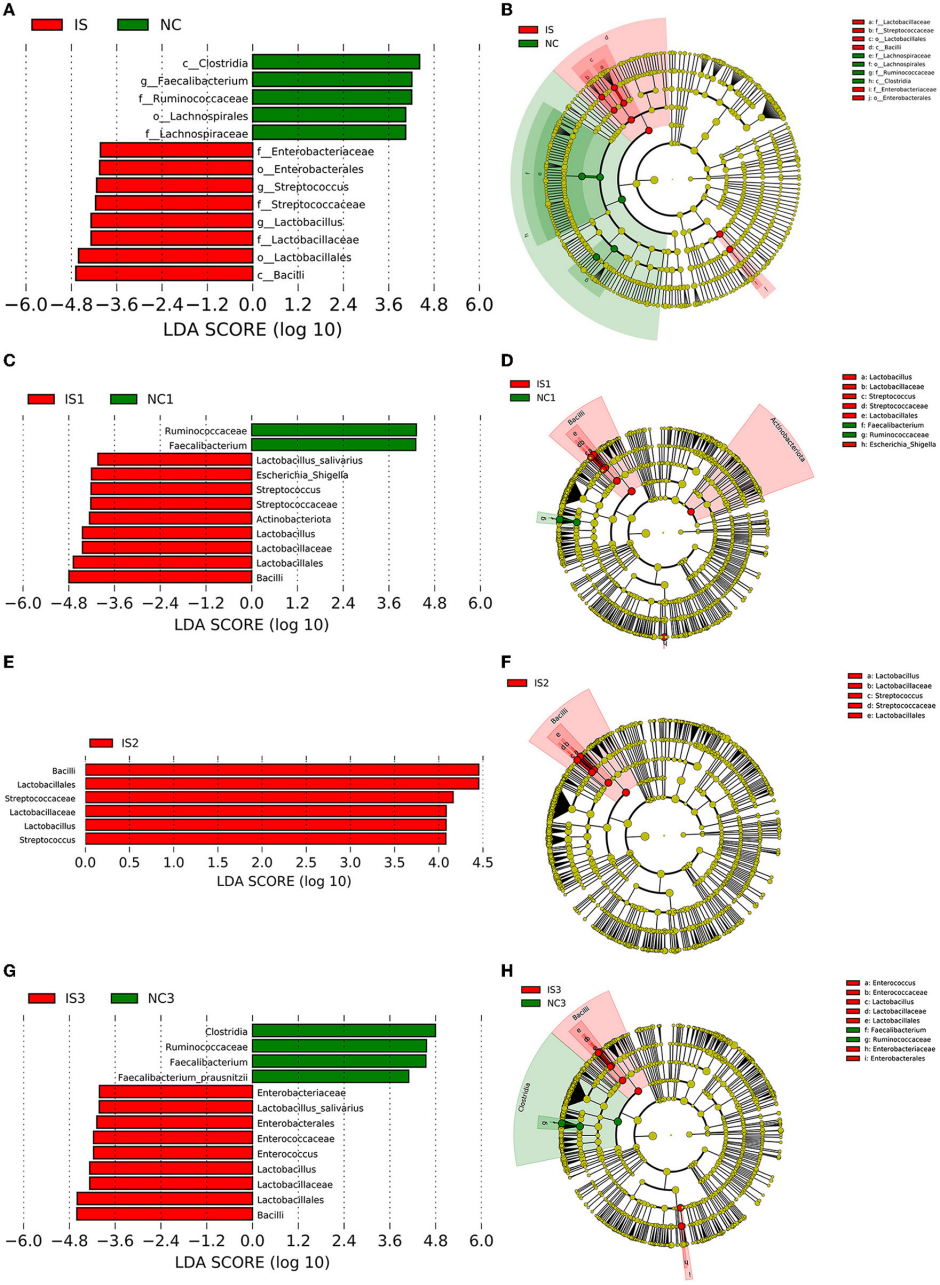
The differences of gut microbiota between normal controls (NCs) and ischemic stroke (IS) patients in the entire population and for each ethnic people(Tujia, Miao, and Han). The IS patients of the Tujia, Miao, and Han people were indicated with IS1, IS2, and IS3, respectively. The NCs of the Tujia, Miao, and Han people were indicated with NC1, NC2, and NC3, respectively. **(A–H)** Histograms of the LDA scores for the bacterial taxa differentially abundant between NCs (green bars) and IS patients (red bars) (LDA score >4.0, *P* < 0.05), and cladograms generated by LEfSe indicating differences in the bacterial taxa between NCs (green dots) and IS patients (red dots) in the entire population and for each ethnic people(Tujia, Miao, and Han).

LDA and LEfSe were used to identify the key gut microbes responsible for distinguishing the IS patients from NCs. In the entire population, 13 differential OTUs were identified (Figure 2A), compared to NCs, patients with IS were characterized by eight enriched OTUs mainly belonging to classes *Bacilli* and *Gammaproteobacteria*, and five depleted OTUs are mainly belonging to class *Clostridia*. *Lactobacillaceae, Streptococcaceae*, and *Enterobacteriaceae families* were more abundant in IS patients, while *Lachnospiraceae* and *Ruminococcaceae* families were more abundant in NCs (Figure 2B). We further assessed the ethnicity-related microbial signatures responsible for distinguishing IS patients from NCs. However, only 11 differential OTUs were identified in the Tujia people (Figure 2C), compared to NCs, patients with IS were characterized by nine enriched OTUs mainly belonging to classes *Bacilli, Gammaproteobacteria, Actinobacteria, Rubrobacteria*, and *Coriobacteriia*, and only two depleted OTUs are belonging to class *Clostridia*. *Lactobacillaceae, Streptococcaceae*, and *Enterobacteriaceae* families were more abundant in IS patients, while only *Ruminococcaceae* family was more abundant in NCs (Figure 2D). And only six differential OTUs were identified in the Miao people (Figure 2E), compared to NCs, patients with IS were characterized by only six enriched OTUs mainly belonging to class *Bacilli* but not depleted OTUs; *Lactobacillaceae* and *Streptococcaceae* families were more abundant in IS patients (Figure 2F). In the Han population, 13 differential OTUs were identified (Figure 2G), compared to NCs, patients with IS were characterized by nine enriched OTUs mainly belonging to classes *Bacilli* and *Gammaproteobacteria*, and four depleted OTUs are mainly belonging to class *Clostridia*. *Lactobacillaceae, Enterococcaceae*, and *Enterobacteriaceae families* were more abundant in IS patients, while only *Ruminococcaceae* family was more abundant in NCs (Figure 2H).

### Traditional risk factors correlate with gut microbiota in ischemic stroke patients

**Figure 3:**
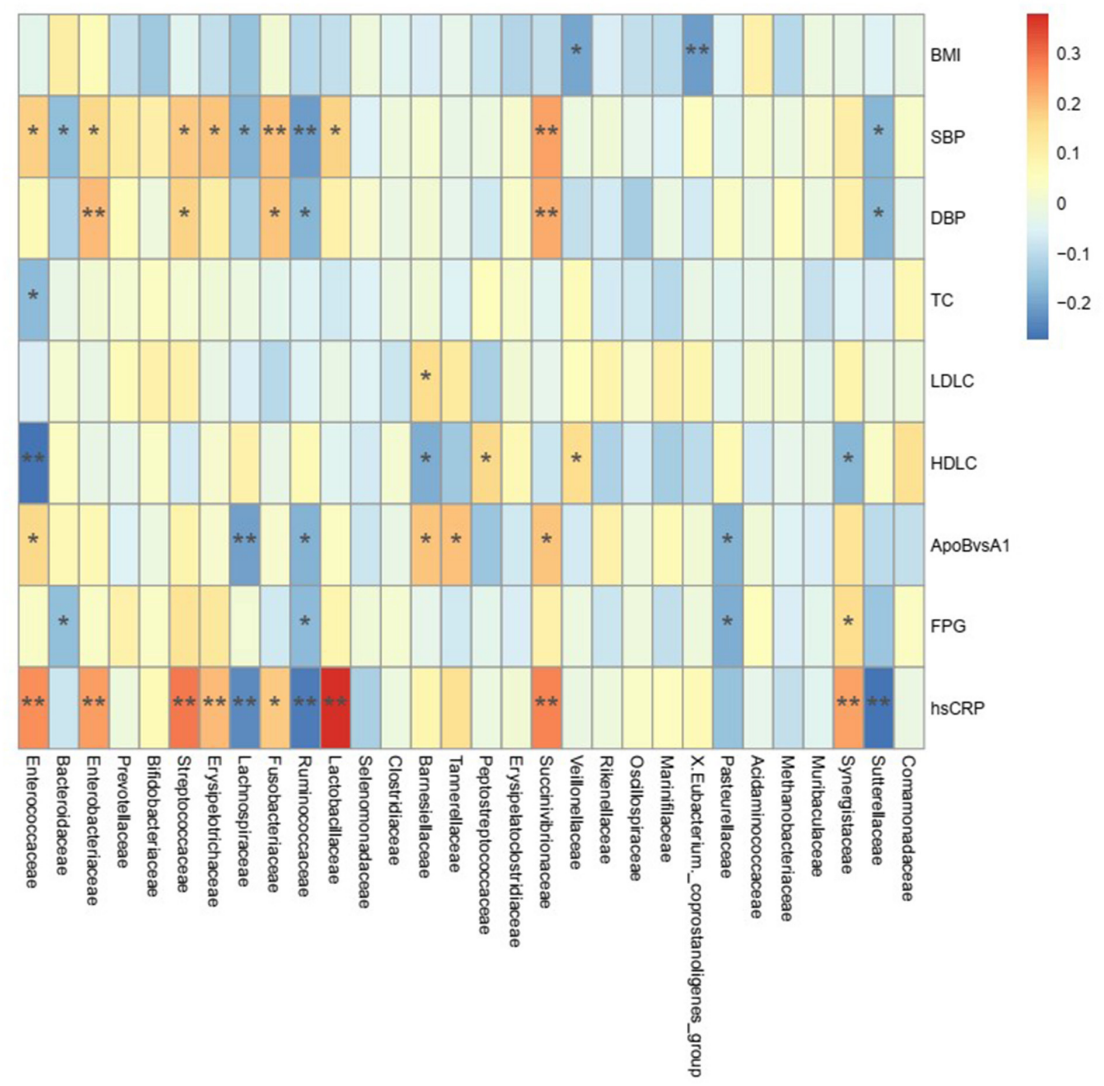
Association between gut microbiota and traditional risk factors of ischemic stroke. The heat map of Spearman's rank correlation coefficients of top 30 gut microbiota in family and 9 traditional risk factors (BMI, SBP, DBP, TC, LDL-C, HDL-C, ApoB/ApoA1 ratio, FPG, and hs-CRP, respectively, just as differences of these risk factors between normal controls and ischemic stroke patients were all statistically significant in Table 1). The correlation effect was indicated by a color gradient from blue (negative correlation) to red (positive correlation). The statistical significance was denoted on the squares (*P < 0.05, **P < 0.01). BMI indicates body mass index; SBP, systolic blood pressure; DBP, diastolic blood pressure; TC, total cholesterol; LDLC, low-density lipoprotein cholesterol; HDLC, high-density lipoprotein cholesterol; ApoBvsA1, apolipoprotein B (ApoB)/ApoA1 ratio; FPG, fasting plasma glucose; hsCRP, high-sensitive C-reactive protein.

To explore the potential associations between gut microbiota and traditional risk factors in IS patients, regardless of ethnicity differences, we constructed a correlation analysis using Spearman's rank correlation coefficients (Figure 3). BMI was negatively correlated with the abundance of families *Veillonellaceae* and *X.Eubacterium, coprostanoligenes_group*. SBP was positively correlated with the abundance of *Enterococcaceae, Enterobacteriaceae, Streptococcaceae, Erysipelotrichaceae, Fusobacteriaceae, Lactobacillaceae*, and *Succinivibrionaceae* families, while negatively correlated with *Bacteroidaceae, Lachnospiraceae, Ruminococcaceae*, and *Sutterellaceae* families, respectively (*P* < 0.05). DBP was positively correlated with the abundance of *Enterobacteriaceae, Streptococcaceae, Fusobacteriaceae*, and *Succinivibrionaceae* families, while negatively correlated with *Ruminococcaceae* and *Sutterellaceae* families, respectively (*P* < 0.05). However, LDL-C was positively correlated with only the abundance of *Barnesiellaceae* family (*P* < 0.05). Moreover, HDL-C was positively correlated with the abundance of *Peptostreptococcaceae* and *Veillonellaceae* families, while negatively correlated with *Enterococcaceae, Barnesiellaceae*, and *Synergistaceae* families (*P* < 0.05). The abundance of *Enterococcaceae, Barnesiellaceae, Tannerellaceae*, and *Succinivibrionaceae* families wase positively correlated, while *Lachnospiraceae, Ruminococcaceae*, and *Pasteurellaceae* families were negatively correlated with ApoB/ApoA1 ratio (*P* < 0.05). FPG was positively correlated with only the abundance of *Synergistaceae* family, while negatively correlated with *Bacteroidaceae, Ruminococcaceae*, and *Pasteurellaceae* families (*P* < 0.05). In addition, hs-CRP was positively correlated with the abundance of *Enterococcaceae, Enterobacteriaceae, Streptococcaceae, Erysipelotrichaceae, Fusobacteriaceae, Lactobacillaceae, Succinivibrionaceae*, and *Synergistaceae*, but negatively correlated with families *Lachnospiraceae, Ruminococcaceae*, and *Sutterellaceae* (*P* < 0.05).

### Additive interaction analysis between traditional risk factors and gut microbiota for first-ever ischemic stroke

To further explore the influence between traditional risk factors and gut microbiota on first-ever IS, the additive interaction analyses (6 × 9 = 54) were performed with six families of gut microbiota and nine traditional risk factors. The LEfSe results showed that six families, *Lactobacillaceae, Streptococcaceae, Enterobacteriaceae, Enterococcaceae, Lachnospiraceae*, and *Ruminococcaceae*, were enriched in NCs or IS patients (Figure 2). Nine traditional risk factors were BMI, SBP, DBP, TC, LDL-C, HDL-C, ApoB/ApoA1 ratio, FPG and hs-CRP, respectively, just as the differences of these risk factors between NCs and IS patients were all statistically significant in Table 1. Only the additive interaction analysis results with the statistical significance were listed in this paper.

As previous studies on microbioal biosphere usually set 0.01% as the relative abundance threshold for rare taxa (Galand et al., 2009; Anderson et al., 2015), and the median of relative abundance of family *Enterococcaceae* was 0.01%, so set 0.01% as the relative abundance threshold for dichotomous variable in this paper. For the interaction analysis, the relative abundance is as follows of family *Enterococcaceae* ≥0.01% = 1 and <0.01% = 0, SBP ≥ 140 mmHg = 1 and <140 mmHg = 0, DBP ≥ 90 mmHg = 1 and <90 mmHg = 0, h-CRP ≥ 5.0 mg/L = 1 and <5.0 mg/L = 0, and OR_00_ = 1. In the R program, the function used a logistic regression model to estimate ORs with 95% CIs. Then, we used the epi.interaction function to calculate additive interactions by means of *RERI, API*, and *SI* with 95% CIs. The results of the interaction analysis showed that there were additive interactions between traditional risk factors (SBP, DBP, and hs-CRP) and family *Enterococcaceae* for first-ever IS. The risks of IS related to SBP, DBP, and hs-CRP were significantly higher in the enriched family *Enterococcaceae* (OR 21.00: 95% CI 7.31–69.67, 27.52: 9.00–99.15, 205.00: 50.81–1187.07, respectively) than in the depleted family *Enterococcaceae* (2.80: 0.93–9.24, 3.82: 1.25–12.39, 29.29: 7.52–153.62, respectively). Although adjusted for age, sex, and ethnicity, the risks of IS related to SBP, DBP, and hs-CRP remained significantly higher in the enriched family *Enterococcaceae* than in the depleted family *Enterococcaceae, ORs* are shown in the Tables 2–4, respectively.

**Table 2:** Additive interaction analysis between SBP and family *Enterococcaceae* for first-ever ischemic stroke.

***Enterococcaceae***	**SBP**	**OR (95% CI)**	**OR (95% CI)^(a)^**	**OR (95% CI)^(b)^**
<0.01%	<140 mmHg	1.00 (Ref.)	1.00 (Ref.)	1.00 (Ref.)
≥0.01%	<140 mmHg	3.69 (1.33–11.53)	3.87 (1.36–12.31)	4.24 (1.46–13.79)
<0.01%	≥140 mmHg	2.80 (0.93–9.24)	3.25 (1.04–11.08)	3.42 (1.09–11.88)
≥0.01%	≥140 mmHg	21.00 (7.31–69.67)	24.18 (8.17–83.06)	26.85 (8.83–95.50)
*RERI* (95% CI)		15.51 (−3.98 to 34.99)	18.06 (−5.19 to 41.31)	20.19 (−6.40 to 46.79)
*API* (95% CI)		0.74 (0.51–0.97)*	0.75 (0.52–0.97)*	0.75 (0.53–0.97)*
*SI* (95% CI)		4.45 (1.57–12.60)*	4.53 (1.63–12.58)*	4.57 (1.68–12.44)*

SBP, systolic blood pressure; RERI, relative excess risk of interaction; API, the attributable proportion due to interaction; and SI, the synergy index. (a) OR was adjusted for age and sex; (b) OR was adjusted for age, sex and ethnicity. *P < 0.05.

**Table 3:** Additive interaction analysis between DBP and family *Enterococcaceae* for first-ever ischemic stroke.

***Enterococcaceae***	**DBP**	**OR (95% CI)**	**OR (95% CI)^(a)^**	**OR (95%CI)^(b)^**
<0.01%	<90 mmHg	1.00 (Ref.)	1.00 (Ref.)	1.00 (Ref.)
≥0.01%	<90 mmHg	5.20 (2.07–14.53)	5.12 (2.02–14.41)	5.70 (2.19–16.55)
<0.01%	≥90 mmHg	3.82 (1.25–12.39)	3.78 (1.24–12.28)	4.16 (1.34–13.82)
≥0.01%	≥90 mmHg	27.52 (9.00–99.15)	27.65 (9.01–99.85)	31.11 (9.87–115.84)
*RERI* (95% CI)		19.50 (−9.01 to 48.02)	19.75 (−9.10 to 48.60)	22.26 (−10.87 to 55.38)
*API* (95% CI)		0.71 (0.41–1.00)*	0.71 (0.42–1.01)*	0.72 (0.42–1.00)*
*SI* (95% CI)		3.78 (1.27–11.20)*	3.86 (1.28–11.67)*	3.83 (1.28–11.43)*

DBP, diastolic blood pressure; RERI, relative excess risk of interaction; API, the attributable proportion due to interaction; and SI, the synergy index. (a) OR was adjusted for age and sex; (b) OR was adjusted for age, sex and ethnicity. *P < 0.05.

**Table 4:** Additive interaction analysis between hs-CRP and family *Enterococcaceae* for first-ever ischemic stroke.

***Enterococcaceae***	**hs-CRP**	**OR (95% CI)**	**OR (95% CI)^(a)^**	**OR (95% CI)^(b)^**
<0.01%	<5.0 mg/L	1.00 (Ref.)	1.00 (Ref.)	1.00 (Ref.)
≥0.01%	<5.0 mg/L	1.82 (0.38–9.83)	1.51 (0.30–8.39)	1.57 (0.31–8.88)
<0.01%	≥5.0 mg/L	29.29 (7.52–153.62)	29.05 (7.33–155.01)	29.44 (7.39–158.26)
≥0.01%	≥5.0 mg/L	205.00 (50.81–1,187.07)	227.25 (54.64–1,363.83)	230.81 (55.21–1,390.98)
*RERI* (95% CI)		174.89 (−117.16 to 466.95)	197.68 (−136.73 to 532.10)	200.80 (−139.70 to 541.30)
*API* (95% CI)		0.85 (0.66–1.05)*	0.87 (0.69–1.05)*	0.87 (0.69–1.05)*
*SI* (95% CI)		7.01 (1.79–27.40)*	7.92 (1.96–32.07)*	7.92 (1.96–31.99)*

hs-CRP, high-sensitive C-reactive protein; RERI, relative excess risk of interaction; API, the attributable proportion due to interaction; and SI, the synergy index. (a) OR was adjusted for age and sex; (b) OR was adjusted for age, sex and ethnicity. *P < 0.05.

## Discussion

In this case–control study, we examined gut microbiome composition between NCs and IS patients among Han, Tujia, and Miao ethnicity, and then analyzed correlations between gut microbes and traditional risk factors. Furthermore, we explored the additive interaction between traditional risk factors and gut microbiota for first-ever IS. We found that in the entire population *Lactobacillaceae, Enterococcaceae, Streptococcaceae*, and *Enterobacteriaceae* families enriched in IS patients; however, *Ruminococcaceae* and *Lachnospiraceae* families enriched in NCs, so the following discussion will focus on these six families enriched in IS patients or NCs.

Furthermore, we found that *Lactobacillaceae, Enterococcaceae, Streptococcaceae*, and *Enterobacteriaceae* families enriched in IS patients were positively correlated with high-risk factors (SBP, DBP, ApoB/ApoA1 ratio, FPG, and hs-CRP) and negatively correlated with the preventive factor (HDL-C) in the IS patients, respectively. However, *Ruminococcaceae* and *Lachnospiraceae* families enriched in NCs were negatively correlated with high-risk factors (SBP, DBP, ApoB/ApoA1 ratio, FPG, and hs-CRP). *Lactobacillus rhamnosus* GG and *Lactobacillus casei* DN-114001 are mostly recognized as probiotics and beneficial microbes (Dietrich et al., 2014); however, increased abundance of family *Lactobacillaceae* was observed among acute IS patients in a Chinese study (Tan et al., 2021). *Lactobacillus* was found to be enriched in the high-risk group compared to the low-risk group in a study of 141 participants aged ≥60 years without prior history of stroke (Zeng et al., 2019). A recent study using a middle cerebral artery occlusion model to explore gut dysbiosis post-stroke in mice revealed a reciprocal relationship between stroke and gut dysbiosis; ischemic stroke rapidly triggers gut microbiome dysbiosis with *Enterobacteriaceae* overgrowth that in turn exacerbates brain infarction (Xu et al., 2021). The interaction analysis also showed that there were additive interactions between traditional risk factors (SBP, DBP, and hs-CRP) and family *Enterococcaceae* for first-ever IS. Few human clinical studies evaluating the efficacy of interventions targeting the gut–brain axis have been published (Vendrik et al., 2020; Yuan et al., 2021). FMT is a novel technique that is beginning to gain traction in the realm of recovery from neurologic injury; however, the lack of human studies on the efficacy of this intervention limits its current applicability in the clinical setting (Panther et al., 2022). Our findings suggest that gut microbiota disturbances could potentially contribute to IS pathogenesis by modulating the host's traditional risk factors, which provides a new avenue by which to understand the prevention of IS.

Consistent with other studies that reported important regional variations in the relative importance of most individual risk factors for stroke may relate to differences in the magnitude of association by ethnic origin (Hyun et al., 2013; Eastwood et al., 2015). Our results also demonstrated that the gut microbiota's taxonomic composition between NCs and IS patients varies in different ethnicity; *Streptococcaceae* family was only more abundant in IS patients of Tujia and Miao people; *Enterococcaceae* and *Enterobacteriaceae* families were only more abundant in IS patients of Han people, and *Ruminococcaceae* family was only more abundant in NCs of Tujia and Han people. This suggests the possibility that regional and ethnic variations in the effect of traditional risk factors for IS may be linked to the differences of gut microbiota in different ethnic people, which suggests the necessity to more deeply understand the ethnic differences of risk factors as they pertain to prevention and treatment of IS.

However, 16S RNA amplicon sequencing represents a comparatively cheap way to measure the relative abundance of microbes present in a sample using next-generation sequencing technology, there are also important limitations. First, 16S sequencing cannot identify novel microbial species nor account for intraspecies variation and mutations as the technique is restricted to genetic reference sequences that must be defined in a database a priori. Second, PCR introduces a bias in 16S tables, as some amplicon sequences will inevitably be amplified more efficiently than others (Acinas et al., 2005). Besides the cheaper cost, because 16S sequencing relies on the 16S subunit ribosomal RNA, which is unique to prokaryotes, most host contamination is not an issue. Also, the data were cross-section rather than longitudinal, and dietary considerations were not included. It would also be interesting to perform dynamic metagenomic and metabolomic analyses to uncover how the gut microbiome and its modulated metabolic pathways dynamically change in IS.

## Conclusion

In conclusion, we first found that gut microbiome were associated with first-ever IS and had ethnic variations of this relationship in the Xiangxi autonomous prefecture, and the gut microbiota disturbances could potentially contribute to IS pathogenesis by modulating the host's traditional risk factors. Race/ethnicity categories may help interpret health-related data and are important in ensuring that prevention programs of IS remain generalizable to diverse populations.

## Data availability statement

The original contributions presented in the study are publicly available. This data can be found in the Genome Sequence Archive in BIG Data Center, Beijing Institute of Genomics (BIG), Chinese Academy of Sciences, under accession numbers CRA006722, the shared URL is https://ngdc.cncb.ac.cn/search/?dbId=gsa&q=CRA006722.

## Ethics statement

The studies involving human participants were reviewed and approved by the Ethics Committees of the Xiangya School of Public Health Central South University (XYGW-2018-26). The patients/participants provided their written informed consent to participate in this study.

## Author contributions

HT, NZ, and HW designed the study. NZ, XWa, MT, YT, QL, CL, and XP recruited participants, collected basic data and samples. NZ and XWu analyzed the data and wrote the manuscript. HT, JD, and XWu contributed to discussion and reviewed/edited the manuscript. HT and XWu supervised the study and the guarantor of this work. All authors read and approved the final manuscript.

## Funding

This study was supported by National Natural Science Foundation of China (81773535); Key Research and Development Program of Hunan Province (2018SK2061); the Fundamental Research Funds for Central Universities of the Central South University (2019zzts328); Scientific Research Project of Hunan Provincial Health Commission (C2014-17, 202212053368); Changsha Municipal Social Science Foundation (kh200502).

## Conflict of interest

The authors declare that the research was conducted in the absence of any commercial or financial relationships that could be construed as a potential conflict of interest.

## Publisher's note

All claims expressed in this article are solely those of the authors and do not necessarily represent those of their affiliated organizations, or those of the publisher, the editors and the reviewers. Any product that may be evaluated in this article, or claim that may be made by its manufacturer, is not guaranteed or endorsed by the publisher.
