# Supplementary material for: Combination effect between gut microbiota and traditional potentially modifiable risk factors for first-ever ischemic stroke in Tujia, Miao and Han populations in China
Source: Front Mol Neurosci. 2022 Oct 25;15:922399. doi: 10.3389/fnmol.2022.922399 (PMC9641726; doi:10.3389/fnmol.2022.922399)
Supplement: Supplementary file 1 [file Data_Sheet_1.pdf]

Supplementary Material

1 Supplementary Figures

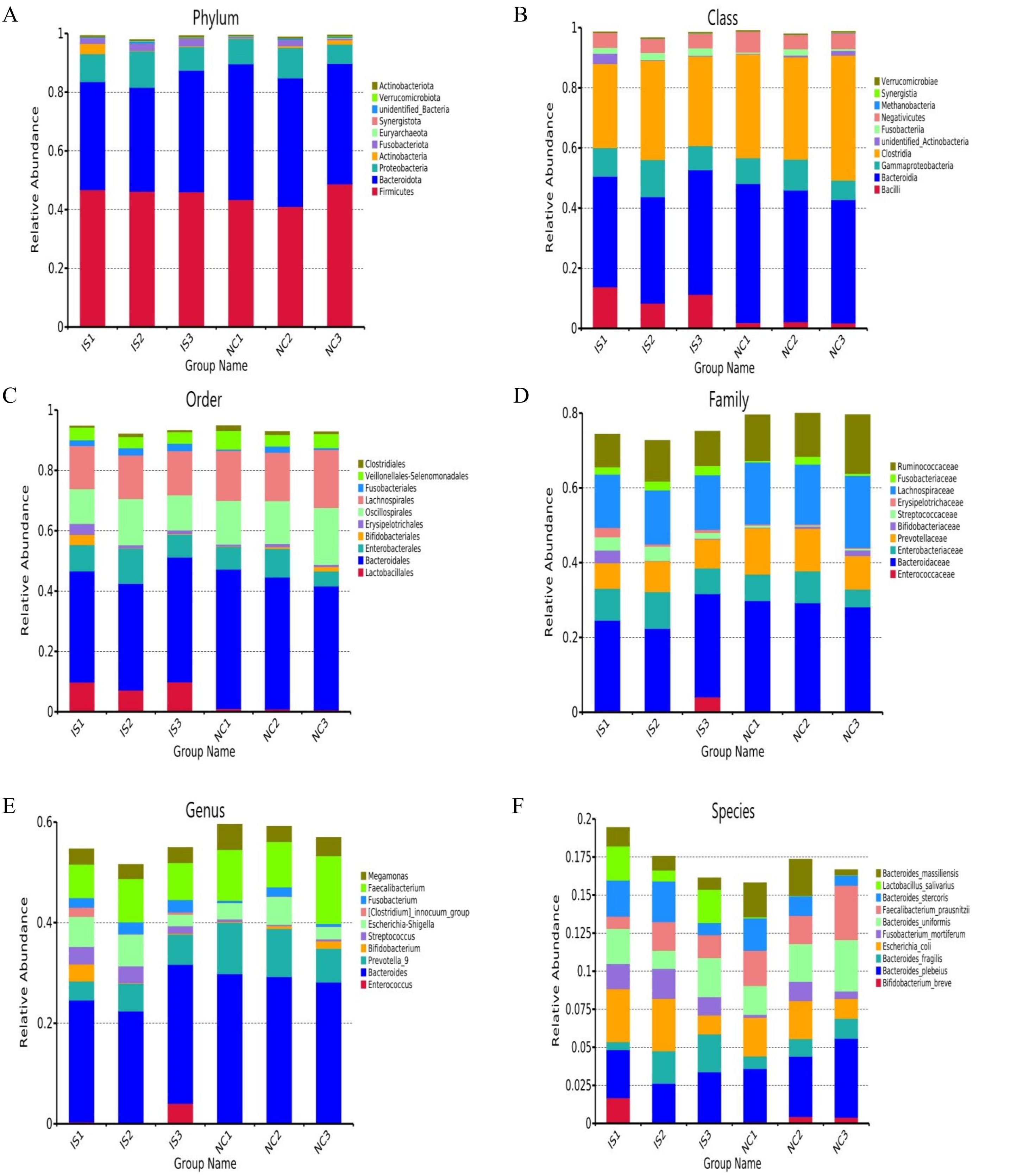

Supplementary Figure 1. Top 10 in relative abundance of gut microbiota (in the level of phylum, class, order, family, genus and species, respectively) between normal controls(NCs) and ischemic stroke(IS) patients among Tujia, Miao and Han ethnicity. The IS patients of the Tujia, Miao and Han people were indicated with IS1, IS2 and IS3, respectively. The NCs of the Tujia, Miao and Han people were indicated with NC1, NC2 and NC3, respectively.

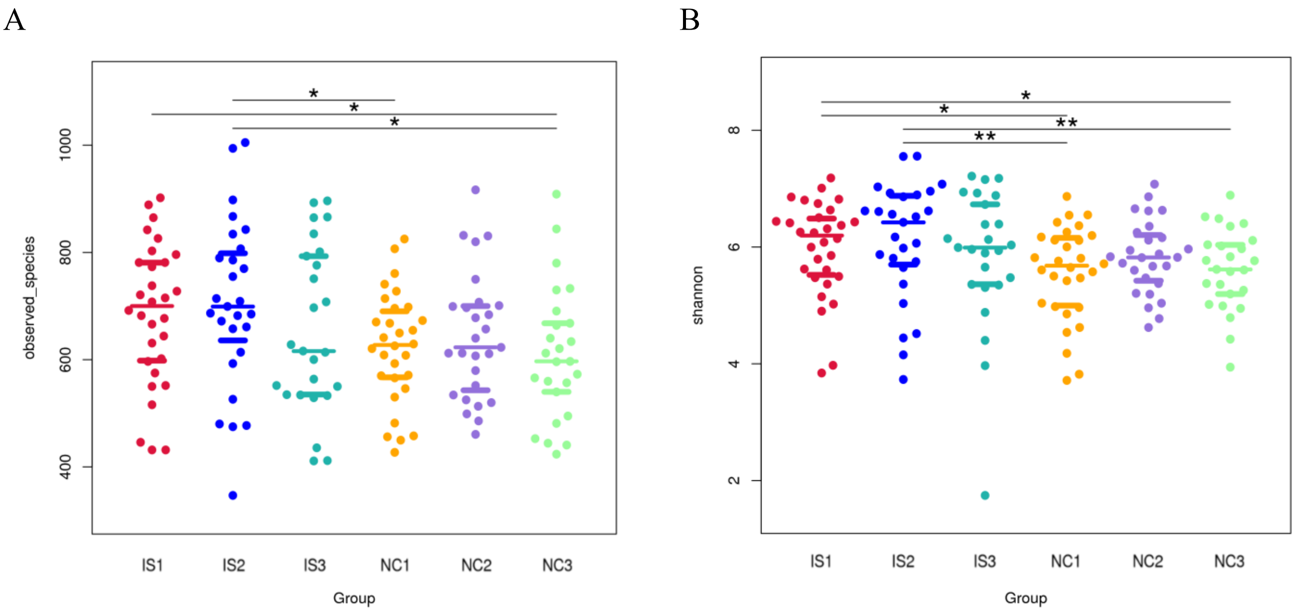

Supplementary Figure 2. Alpha diversity indices for normal controls(NCs) and ischemic stroke(IS) patients among Tujia, Miao and Han people, observed_species(A) and shannon(B). **P*<0.05, ***P*<0.01, Wilcoxon rank sum test. The IS patients of the Tujia, Miao and Han people were indicated with IS1, IS2 and IS3, respectively. The NCs of the Tujia, Miao and Han people were indicated with NC1, NC2 and NC3, respectively.
